# Assessing the Correct Documentation of Time and Physician Information on Medical Records in the Emergency Department of Queen's Hospital: An Audit and Re-audit

**DOI:** 10.7759/cureus.33000

**Published:** 2022-12-27

**Authors:** Anastasia Gkiala

**Affiliations:** 1 Emergency Medicine, Queen's Hospital, Barking, Havering and Redbridge University Hospitals NHS Trust, London, GBR

**Keywords:** signature, name, gmc number, time, date, emergency department, documentation, medical records

## Abstract

Background: Medical records are confidential medical and legal documents describing a patient’s contact with a healthcare facility. The quality of documentation has been found to be lower in settings of high patient volume and complex cases, such as the emergency department (ED). The variety and number of healthcare professionals involved in the care of the patient also negatively affect the quality of documentation. The aim of this paper is to present the results of an audit and re-audit conducted in the ED of Queen's Hospital, Romford, to assess ED record documentation against General Medical Council (GMC) and Royal College of Physicians (RCP) standards.

Methods: For the audit, all records of patients who were discharged from the ED of Queen's Hospital in one day were reviewed and evaluated on whether they have a date, time, the full name of the physician, their GMC number, and signature documented, as per GMC and RCP official guidelines. No medical information or patient data were recorded. After the implementation of the change aiming to raise awareness of ED staff, a new sample was collected two months later, and the same parameters were assessed against the set standards.

Results: Results of the audit showed a low percentage of documentation of all parameters, especially of GMC number and signature. After the presentation of the results and implementation of change, the results of the re-audit demonstrated significant raise in all percentages, with a relative improvement of 40% regarding the recording of GMC number and 65% regarding signature. However, the documentation of these two parameters remained low and below acceptable levels.

Discussion: The re-audit results underline that the low compliance was significantly improved after the implementation of measures aiming to increase correct documentation awareness among ED staff. However, to maintain and even raise the level of current practice, additional systematic measures need to be put into action.

## Introduction

Medical records are confidential medical and legal documents describing a patient’s contact with a healthcare facility. They are very important for future reference and clear communication between doctors who wish to have a better understanding of the patient’s condition and clinical management, both in a hospital environment and in the community [1,2]. The importance of clear documentation becomes even more vital during periods of patient care transition, when low-quality patient notes may result in unnecessary delays and medical errors [3,4]. Correctly written medical records ensure that the patient’s assessed needs are met comprehensively, and they can also provide valuable evidence when legal use is appropriate [5]. Moreover, given that patient records are a considerable source of information for funding applications, resource allocation, and research and quality improvement projects organisation, inaccurate or incomplete documentation may negatively affect the quality of healthcare services indirectly related to clinical care [4,6].

Medical records should include information about the patient’s clinical presentation, past medical history, physical examination, and actions taken towards diagnostic examinations and treatment. A chronologically coherent, clear, and accurate medical record documentation is of uttermost importance, and the exact date and time of events must be clearly stated as well as the name of the responsible physician, their General Medical Council (GMC) number, and their signature [7-9].

Existing literature underlines the negative impact poor documentation can have on patient care and healthcare planning. One retrospective study conducted in 2018 showed an inverse correlation between the accuracy of patient records and overall antibiotic order resolution time [10]. In another retrospective chart review of patients who had undergone carotid endarterectomy, the appropriate scheduling of the procedure was affected by a lack of clear documentation [4].

Despite their widely accepted importance, medical notes are frequently viewed as low priority by doctors. It is not uncommon for notes to have missing information, illegible writing, inconsistencies, and even inappropriate comments [11]. Quality has been found to be negatively affected by patient volume, the complexity of cases, and the variety and number of healthcare professionals involved in the care of the patient [12,13]. This association is particularly evident in the emergency setting, where the high number of doctors involved, the intense medical activities taking place under time pressure, overcrowding, and frequent interruptions can have a major adverse effect on correct and complete documentation [14-16].

The introduction of electronic medical record (EMR) systems in numerous emergency departments (EDs) aimed to improve quality and clarity and reduce medical documentation emissions and errors. While being a very promising development, opinions regarding the efficacy of this novel documentation method are still conflicting [17-19].

## Materials and methods

Audit

The Aim of the Audit

The aim of this audit was to identify if paper medical record documentation in the ED of Queen's Hospital, Romford, United Kingdom was conducted as per the GMC and Royal College of Physicians (RCP) standards. According to these standards, the precise date, time, name, GMC number, and signature of the responsible physician should be noted as these are crucial for maintaining the best medical practice and communication among doctors. Depending on the compliance of medical staff with the above standards, suggestions of improvement measures and their implementation would follow aiming to improve clinical practice as much as possible by the end of January 2022.

Patient and Physician Consent

This audit did not focus on the content of the notes, but on the clear documentation of the date, time, name of the doctor, and their GMC number and signature. For this reason, no patient consent was obtained since no patient information was included in the data collection. In addition, the name, GMC number, and signature of the authoring physician were not recorded, only their presence or absence on the medical record was noted. For this reason, no authoring physician consent was obtained.

Data Collection

For the conduction of the audit, all paper records of patients who were discharged from the ED in Queen's Hospital on 22 November 2021 were reviewed the following morning, on 23 November 2021. The records were evaluated on whether they had a date, time, full name of the physician, and their GMC number and signature documented. A total of 117 discharge notes were assessed. Of them, 34 had no entries, being either direct referrals to specialities or no-shows. Consequently, 83 medical records were evaluated. No medical information or patient or physician data were recorded.

Standards

The data collected from the ED records were compared against the standards described below.

GMC: “Using your registered name and number” - “It's good practice to display your registered name and GMC reference number at your place of work, on your professional website, in medical records, in letters, emails and reports that you write”.

RCP: “Generic medical record keeping standards” - “Every entry in the medical record should be dated, timed (24-hour clock), legible and signed by the person making the entry. The name and designation of the person making the entry should be legibly printed against their signature”.

Audit Criteria

The acceptable percentage of evaluated ED notes with clear documentation of the date and exact time of the medical record entry was set a priori to 90%. The same percentage (90%) was the acceptable cut-off for ED records where the name, GMC number, and clinician signature were clearly documented.

Data Analysis

The data were collected and inserted in a Microsoft Excel document (Microsoft Corporation, Redmond, WA). Statistics were also performed using Microsoft Excel.

Intervention

The results of the audit were presented to the ED Junior Doctor Teaching on 27 January 2022. In addition to the results, suggestions for improvement of current practice were introduced to the ED doctors.

**Figure 1:**
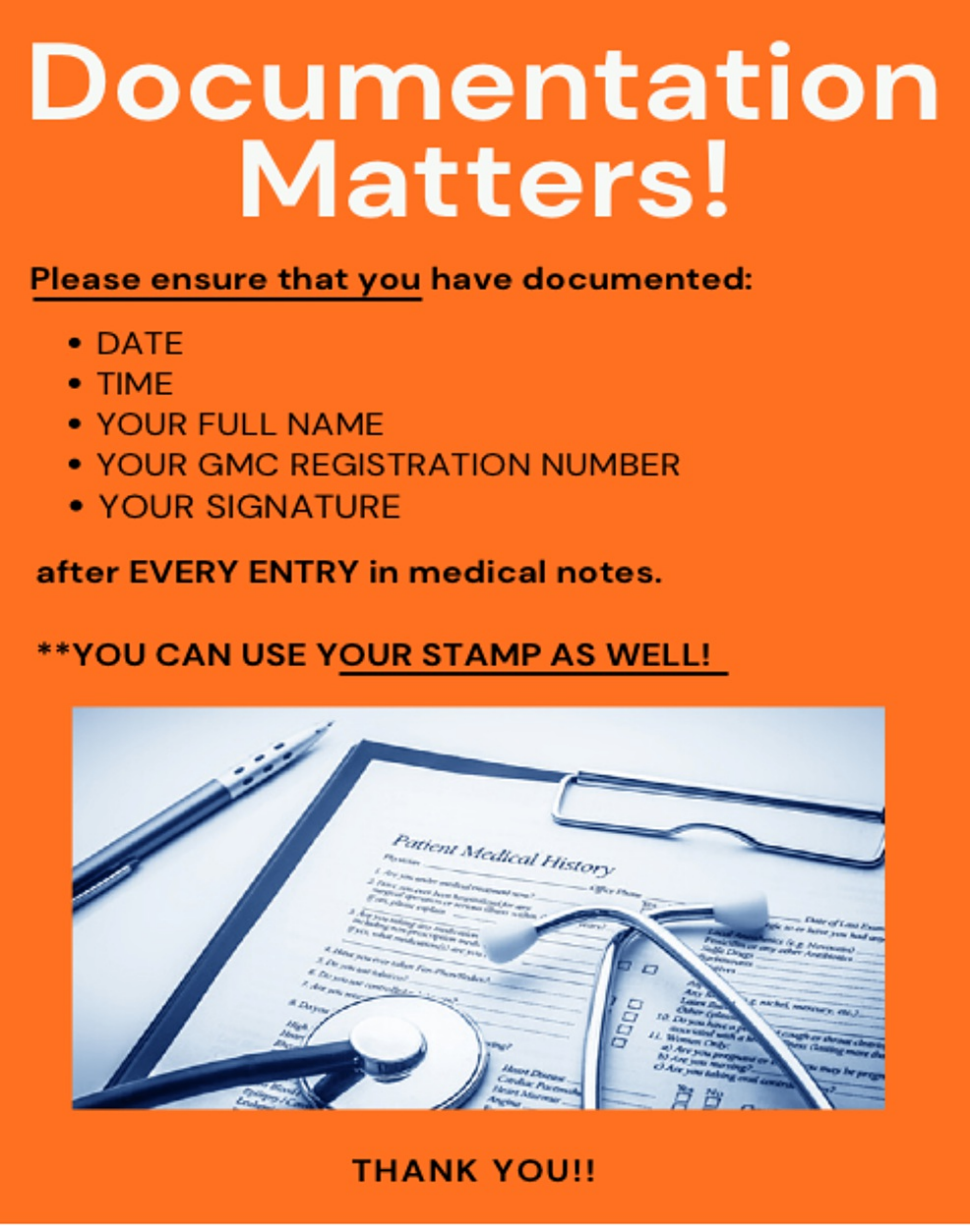
Poster posted above the doctors’ desks in various ED areas GMC: General Medical Council.

These recommendations, implemented on 29 November 2021, included reminder emails circulated within the ED staff. The emails underlined the documentation information that should be included in every ED record entry and encouraged ED staff to comply with the standards. In addition, reminder posters were created and posted on the wall of the doctor’s desks in the ED areas of Queen's Hospital, namely, Majors A, Majors B, Paediatric ED, and Resus. The content of the posters was clear and similar to that of the emails, namely, bullet points reminding doctors to document the date, time, and their name, GMC number, and signature at the end of every entry and encouraging them to use their personal stamp, which includes their name and GMC number (Figure 1).

Re-audit

The Aim of the Re-audit

The aim of the re-audit was to determine if suggestions implemented after the presentation of the audit results have contributed to the improvement of current practice two months after the audit was conducted.

Data were collected in the exact same way as in the initial audit. No patient or physician consent was obtained since no clinical or identification details were recorded.

For the re-audit, which was conducted two months after the initial audit, all records of patients who were discharged from the ED of Queen's Hospital on 18 January 2022 were reviewed for the same documentation parameters. A total of 123 discharge notes were assessed. Of these, 32 had no entries, being either direct referrals to specialities or no-shows. As a result, 91 medical records were evaluated.

Data analysis was completed in the same way as for the audit. Further analysis was conducted using RStudio (version 2022.12.0+353; RStudio, Boston, MA). Each parameter was analysed separately using the chi-squared test.

## Results

Audit

Statistical analysis of the 83 ED medical records collected for the audit was conducted. Results showed that most of the medical notes had clear documentation of the date and time of the entry, although still not reaching acceptable audit criteria. This was just below 90% of the overall records for date and slightly lower (84.3%) for time. When assessing the documentation of the physician's name at the end of the entry, this was recorded by approximately 82% of notes. The compliance with established standards was significantly lower when documentation of the physician's GMC number and signature was evaluated. The percentage for those two parameters was just below 40%, equal to the GMC number and signature (Table 1).

**Table 1:** Summary of the audit results GMC: General Medical Council.

Parameter documented	Number of notes (out of 83)	Percentage
Date of entry documented	74	89.2%
Time of entry documented	70	84.3%
Full name of physician documented	68	81.9%
GMC number of the physician documented	32	38.6%
Signed by physician	32	38.6%

Re-audit

The results of the statistical analysis of the ED notes evaluated at the re-audit two months after the completion of the audit were the following. From a total of 91 ED medical records, the percentage of those with the clearly recorded date and time increased to just below 95%, equal for the two parameters. Almost 90% of ED notes had the full name of the physician recorded, while in half of them, the doctor’s GMC number was clearly documented. The percentage of the notes signed was approximately 60% (Table 2).

**Table 2:** Summary of the re-audit results GMC: General Medical Council.

Parameter documented	Number of notes (out of 91)	Percentage
Date of entry documented	85	93.4%
Time of entry documented	85	93.4%
Full name of physician documented	80	88.8%
GMC number of the physician documented	45	49.5%
Signed by physician	53	58.2%

The statistical level of significance between the number of records documenting the aforementioned parameters was set to 0.05 and evaluated using the chi-squared test for each parameter. More specifically, the increased number of medical notes with documented date, time, name, and GMC number was not statistically significant (p > 0.05). However, there was a statistically significant difference between the medical notes signed by the authoring physician before and after the implementation of the change (p < 0.01).

## Discussion

The ED can often be a stressful, busy place, where critically ill patients need to be managed quickly and efficiently. Time is pressing, and a multidisciplinary approach is usually mandatory, sometimes involving a wide spectrum of different healthcare professionals completing the same medical record. In such an intense environment, correct documentation can sometimes be neglected by being regarded as secondary or of low significance. Nevertheless, it should become evident that accurate medical documentation, especially in an ED setting, is not only essential for correct patient management and clear communication between healthcare professionals but can also act as valuable legal evidence when needed.

Significantly low compliance with the GMC and RCP standards was observed after analysis of the audit results regarding clear documentation of date, time, and personal/professional information of the responsible physician on ED medical notes. It became evident that further efforts to improve the quality of ED records were necessary. The results of the audit were presented, and measures aiming to improve current practices were readily implemented. Almost two months after the implementation of the change, better compliance with the GMC and RCP standards was observed regarding clear documentation of all five parameters tested. This improvement, although still not reaching the acceptable percentage of compliance, reached 25% in terms of documentation of the GMC number and 50% when the signature of the responsible physician was tested. The increased signature documentation was found to be statistically significant (p < 0.05).

These findings are consistent with the existing literature. A systematic review conducted in 2018 by Lorenzetti et al. underlined audit/feedback, regular reminders, templates, and multi-pronged education interventions as promising means of improving the quality of documentation in the ED. These measures were considered to be particularly useful in settings without EMR systems [20]. The use of audit, reminders, and a standardized approach (e.g. stamping instead of handwriting name and GMC number) played a primary role in attempting to improve practice in our audit. Similar audits conducted in a non-ED environment supported active (e.g. audit/feedback or templates) and/or resourceful interventions aiming to actively engage physicians as more effective educational means compared to passive interventions (e.g. printed education materials) [15]. As per Callen et al., another key to the success of any intervention is its adaptability and smooth integration into the existing system, as well as its wide acceptance from all stakeholders [21].

The introduction of EMRs has been adopted by many hospitals in the UK and has undoubtedly many advantages [22]. However, many EDs still rely on paper documentation, a fact that further supports the usefulness of this audit.

The results of the re-audit, although better after the implementation of change, still have room for improvement to reach the acceptable level of 90% or fully comply with official guidelines. Limitations of the study included the short period of data collection and the fact that one physician usually authored more than one set of notes within the set timeframe, enhancing the positive or negative effect on results. On the other hand, the samples may be considered representative as they included all the notes of patients who attended ED that particular weekday (no weekend day was assessed), both morning and night, authored by doctors of different levels of seniority. However, the doctors authoring the notes were not the same between the audit and the re-audit. This fact does not support the comparability of the two samples, although, as a standard, each shift is comprised of a similar number and senior-to-junior doctor ratio, although these numbers were not recorded in the audit.

To maintain and even raise the level of current practice, systematically implemented measures may need to be implemented. One suggestion could be the introduction of the topic of correct and complete medical record keeping in the department induction of all new ED staff members and their active education on that matter. This could be achieved through an interactive, e-learning platform or even through interactive face-to-face simulations.

## Conclusions

The audit described above assessed documentation of date, time, and personal/professional information of the responsible physician on ED medical notes in Queen's Hospital, Romford, United Kingdom and demonstrated poor compliance with official GMC and RCP guidelines in all five parameters tested. The audit results were presented to the department and action was taken to raise awareness of the issue and improve clinical documentation. The re-audit conducted two months later showed improved compliance with standards, which was statistically significant when the documentation of the doctor's signature was assessed. Although percentages of GMC numbers and signature documentation improved, they remained low (49.5% and 58.2%, respectively).
